# Boswellic acids extract attenuates pulmonary fibrosis induced by bleomycin and oxidative stress from gamma irradiation in rats

**DOI:** 10.1186/1749-8546-6-36

**Published:** 2011-09-30

**Authors:** Eman Noaman Ali, Somaya Zakaria Mansour

**Affiliations:** 1 Radiation Biology Department, National Centre for Radiation Research and Technology, Atomic Energy Authority, Cairo, Egypt

## Abstract

**Background:**

Interstitial pulmonary fibrosis is characterized by an altered cellular composition of the alveolar region with excessive deposition of collagen. Lung inflammation is also common in pulmonary fibrosis. This study aims to test the inhibition of 5-lipooxygenase (5-LOX) by boswellic acid (BA) extract in an experimental model of pulmonary fibrosis using bleomycin (BL).

**Methods:**

Boswellic acid extract (1 g/kg) was force-fed to rats seven days prior to administration of BL or gamma irradiation or both. BL (0.15 U/rat) in 25 μl of 0.9% normal saline (NS) or 0.9% NS alone was administered intratracheally. Rats were exposed to two fractionated doses of gamma irradiation (0.5 Gy/dose/week) with a gamma cell-40 (Cesium-137 irradiation units, Canada) during the last two weeks of the experiment. BA was administered during BL or irradiation treatment or both. After the animals were sacrificed, bronchoalveolar lavage was performed; lungs were weighed and processed separately for biochemical and histological studies.

**Results:**

In rats treated with BL, levels of transforming growth factor-β1 (TGF-β1) and tumor necrosis factor-α (TNF-α) were significantly elevated (*P *= 0.05 and *P *= 0.005). Hydroxyproline was highly and extensively expressed. Immunoreactive compounds were abundantly expressed, represented in the levels of macrophages infiltrate, accumulation of eosinophils and neutrophils in the lung as well as the aggregation of fibroblasts in the fibrotic area. The levels of lipoxygenase enzyme activity were significantly increased (*P *= 0.005). Antioxidant activities measured in BL-treated rats deteriorated, coupled with the elevation of both levels of plasma lipid peroxide (LP) content and bronchoalveolar lavage lactate dehydrogenase activity. BA-treated rats had reduced number of macrophages, (*P *= 0.01), neutrophils in bronchoalveolar lavage (*P *= 0.01) and protein (*P *= 0.0001). Moreover, the hydroxyproline content was significantly lowered in BA-treated rats (*P *= 0.005). BA extract inhibited the TGF-ß induced fibrosis (*P *= 0.01) and 5-LOX activity levels (*P *= 0.005).

Histologically, BA reduced the number of infiltrating cells, ameliorated the destruction of lung architecture and attenuated lung fibrosis.

**Conclusion:**

BA attenuates the BL-induced injury response in rats, such as collagen accumulation, airway dysfunction and injury. This study suggests that the blocking of 5-LOX may prevent the progression of fibrosis.

## Background

There is no effective treatment to prevent or reverse lung fibrosis, the process of which is poorly understood [1]. Idiopathic pulmonary fibrosis (IPF) is one of the most common chronic interstitial lung diseases with a mortality rate of up to 70% five years after diagnosis; most therapeutic strategies have been based on eliminating or suppressing the inflammatory component without evidence of efficacy [2]. A recent clinical trial of long-term treatment with IFN-1b on IPF found some effects on mortality; however, no impact on the process of fibrogenesis was observed [3].

Transforming growth factor ß1 (TGF-ß1), among a series of cytokines and chemokines, has been related to the initiation and progression of fibrosis [4,5]. TGF-ß1 promotes myofibroblast proliferation, induces the synthesis of extracellular matrix (ECM) proteins and inhibits ECM degradation by inducing antiproteinases or reducing metalloproteases [6]. Transient (7-10 days) overexpression of active TGF-ß1 by adenoviral vector gene transfection in rat lungs induces a severe and progressive fibrosis [7]. Moreover, the blocking of TGF-ß1 in animal models, such as with the use of soluble type II receptors for TGF-ß1 [8], may be effective in reducing fibrosis.

Inflammatory cytokines play an important role in the initiation and perpetuation of the fibrotic process. Tumor necrosis factor (TNF)-α is a proinflammatory cytokine with many biological properties [9] and is critical in the development of pulmonary fibrosis [10]. Anti-TNF-α antibody attenuates BL-induced pulmonary fibrosis in mice [11]. In addition, a soluble receptor for TNF-α alleviates BL-induced pulmonary fibrosis [12].

TNF-α receptor knockout mice are protected against pulmonary fibrosis caused by silica, BL and asbestos [13-15]. Thus, TNF-α in conjunction with BL may accentuate the fibrotic process. Since TNF-α overexpression alone does not induce pulmonary fibrosis [16], TNF-α overexpression may sensitize the rats to fibrotic agents. Pulmonary fibrosis can be induced in rats by several means including BL, herpes virus (an adenovirus) expressing TGF-β1, silica, asbestos, butylated hydroxytoluene and oxygen or irradiation. BL, which is a chemotherapeutic drug used to treat cancer and can cause pulmonary fibrosis as a complication, has been used in research to induce pulmonary fibrosis in mice and rats and produce an oxidative injury resulting in DNA damage and the destruction of alveolar epithelial cells [11]. In addition, another type of fibrotic agent, namely radiation exposure, can directly produce oxidant injury and DNA damage [17]. TGF-β1 is a well known stimulant of extracellular matrix production by fibroblasts and has been suggested to play an important role in the development of pulmonary fibrosis [18,19].

The gum resin of *Boswellia serrata*, a kind of deciduous tree found in parts of China and India has long been used for treating inflammatory and arthritic diseases [20]. Boswellic acids (BA), which are pentacyclic triterpenic acids, found in the gum resin of the tree are responsible for its anti-inflammatory properties [21,22]. Suppression of leukotriene synthesis *via *inhibiting 5-lipoxygenase is considered the main mechanism underlying their anti-inflammatory effects. BAs are specific, non-redox inhibitors of 5-lipoxygenase as they do not affect 12-lipoxygenase and cyclooxygenase (COx) activities [22-24]. Moreover, BA inhibit leukocyte elastase for anti-inflammation [25,26].

Pulmonary fibrosis is a debilitating condition for which there is no effective therapy and patients' prognosis is poor [27]. Treatment of pulmonary fibrosis has been based primarily on anti-inflammatory and immunosuppressive therapies with very limited success [28]. As such, new therapeutic strategies are much needed. BA fraction is an agent with demonstrated anti-fibrotic activity in several organs in animals, including the lung [29]. A phase II clinical study shows BA to be a promising treatment for idiopathic pulmonary fibrosis [30]. BA may be beneficial for a range of fibrotic conditions through both anti-inflammatory and anti-fibrotic mechanisms. Although the routes of exposure differ between the intratracheal rats' model and systemic delivery to the lung as occurs in humans with BL lung toxicity, the resulting patchy interstitial fibrosis is similar in the two situations [31].

TGF-ß1 may be a key growth factor in the initiation of fibrosis [32]. Overexpression of TGF-ß1 by administering BL to rats induces potent and progressive pulmonary fibrosis [33]. It is possible to decrease BL-induced lung fibrosis by inhibiting TGF-ß1 type II receptor [34] or TGF-ß1 neutralizing antibodies [35], or inhibiting TGF-ß pathways, through a negative regulator of TGF-ß1 signaling [36]. Therefore, therapeutic strategies to inhibit TGF-ß1 by interfering with the receptor signaling process appear to be the right intervention to prevent the initiation of fibrosis.

However, BA may target the parenchymal cells that respond to TGF-ß1. BA are specific and potent inhibitors of TGF-ß1 signaling *in vivo *[37]. Moreover, preliminary investigations with this extract show considerable attenuation of BL-induced lung fibrosis when administered simultaneously with BL [38].

Tumor necrosis factor alpha (TNF-α) is important in the development of pulmonary fibrosis. Since chronic overexpression of TNF-α alone did not produce pulmonary fibrosis, we hypothesized that chronic overexpression of TNF-α might make the lungs more susceptible to BL or TGF-β1 [39].

While TNF-α is reported to be a major cytokine for the development of pulmonary fibrosis, recent studies show that TNF-α both induces and protects against disease processes [40]. Kuroki *et al*. demonstrates that TNF-α lessened pulmonary inflammation in TNF-deficient mice by inducing apoptosis of infiltrating inflammatory cells [41]. With respect to pathogenesis of lung diseases, TNF-α may be responsible for several lung diseases such as pulmonary fibrosis, acute lung injury and pulmonary emphysema [42-44].

The inflammation major component in the pathogenesis of interstitial lung disease that is orchestrated in part by endogenous and migrating leukocytes [45]. These leukocytes together with lung epithelial and endothelial cells create a feedback loop where stimuli from injury responses can activate alveolar and interstitial macrophages [46]. This study aims to test the inhibiton of 5-LOX by BA extracts in an experimental model of pulmonary fibrosis using BL.

## Methods

### Materials and instrument

The oleogum resin of *Boswellia carterii *Birdwood (Bursearceae) was purchased from a local market. All chemicals and reagents used in the experiment were of analytical grade and purchased from Merck (Germany) and Sigma Aldrich Chemie (Germany). Assay kits for testing TGF-β1 and TNF-α ELISA were purchased from Dynatech Laboratories (USA), assay kit of 5-lipooxygenase activity from Cayman Chemical Company (USA) and lactate dehydrogenase (LDH) activity kit from Sigma Aldrich Chemie (Germany).

### Experimental animals

Male Swiss albino rats (100-110 g) were obtained from the Egyptian Organization for Biological Product and Vaccines in Giza, Egypt and were used throughout the present experiments. The animals were housed in cages in a climate-controlled room with 55% of humidity at 25°C and a 12-hour light/dark cycle. Rats were fed on standard pellets of rat diet (PMI Feeds, Egypt) and water *ad libitum*. The animals were allowed to acclimatize to the environmental conditions for one week before experiments. All animal handling procedures were approved by the Ethics Committee of the National Centre for Radiation Research and Technology, Atomic Energy authority, Cairo, Egypt and in accordance with the recommendations for the proper care and use of laboratory animals (NIH publication No.85-23, revised 1985).

### Gamma radiation procedure

Irradiation of animals was carried out at the National Centre for Radiation Research and Technology (NCRRT) in Cairo, Egypt, with a gamma cell-40 (Cesium-137 irradiation units, Canada). The irradiation dose rate was 0.61 Gy/minute. Animals (whole body) were exposed to two fractionated doses (0.5 Gy/dose/week) during the final two weeks of the experiments.

### Induction of lung fibrosis

To induce pulmonary fibrosis, we treated the rats with 0.15 U BL in 25 μl 0.9% normal saline (NS) or 0.9% NS alone *via *intratracheal administration according to the method described by Cortijo *et al*. [47].

### Preparation of boswellic acid (BA) fraction

The oleo gum resin (1 kg) was extracted with methylene chloride (5 L). The extract was concentrated under reduced pressure to yield 250 g of semisolid oleoresin. The extract was then further fractionated. Briefly, the semisolid oleoresin was dissolved in petroleum ether and the soluble fraction was discarded (95%) and non-soluble fraction (terpenoid portion) (5%) was re-dissolved in methanol and applied on a thin layer chromatography (TLC) with a solvent system of chloroform and methanol (9:1). The TLC showed four bands of BA fractions, consistent with the report by Badria *et al*. [48]. The fractionation of the oleogum resin of Boswellia resulted in the isolation and identification of nine compounds, namely palmitic acid and eight triterpenoids belonging to lupane, ursane, oleanane and tirucallane skeleta isolated from the resin. These triterpenoids are lupeol, beta-boswellic acid, 11-keto-beta-boswellic acid, acetyl beta-boswellic acid, acetyl 11-keto-beta-boswellic acid, acetyl-alpha-boswellic acid, 3-oxo-tirucallic acid and 3-hydroxy-tirucallic acid. Rats were orally administrated with the BA fraction (1 g/kg body weight/day) dissolved in distilled water for 28 days starting from seven days before the BL instillation.

### Animal groups

The experimental animals were divided into eight groups (*n *= 10), namely (1) Control: healthy animals receiving saline; (2) BA: animals receiving the BA fraction; (3) IR: animals exposed to γ irradiation; (4) BA + IR: animals receiving the BA fraction and exposed to γ irradiation; (5) BL: animals injected with BL; (6) BA + BL: animals receiving the BA fraction and injected with BL; (7) BL +IR: animals injected with BL and exposed to γ irradiation; (8) BA + BL+ γ irradiation: animals receiving the BA fraction and injected with BL and exposed to γ irradiation. Rats of all groups received the last irradiation exposure on the day prior to overnight fasting and sacrifice. Blood samples were collected by heart puncture. Plasma of each blood sample was separated and kept frozen for biochemical assays. Bronchoalveolar lavage was performed and lungs were weighed and processed separately for biochemical and histological studies. Lung samples were kept at -80°C until biochemical assays.

### Biochemical assays

#### Bronchoalveolar lavage

For the determination of inflammation following BL administration, each animal was killed with diethyl ether; lungs and trachea were removed. Two successive 5.0 ml aliquots of warm (37°C) phosphate-buffered saline (PBS, pH7.4) were infused and slowly withdrawn from the lungs through a cannula inserted into the trachea. Recovered bronchoalveolar lavage fluid (BALF) volumes routinely measured between 8.0 and 9.0 ml. BALF were centrifuged at 1000×*g *for 10 minutes (Janetzki, Model T30, Germany) at 4°C, after which the supernatants were removed and saved for assays of lactate dehydrogenase (LDH) and protein content. The cell pellets were resuspended in PBS and total cell counts were determined on a hemocytometer. Concurrently, alveolar macrophage viabilities were assessed by trypan blue exclusion. Furthermore, BALF cell samples were centrifuged and stained with Wright's stain and differential cell counts determined under a light microscope by counting 300 to 400 cells per animal. Absolute cell numbers were log_10 _transformed for data analysis and presentation according to methods by Cortijo *et al*. [47]. LDH activities of the BALF supernatants were determined with a commercially available kit. BALF protein content was determined according to the method of Lowry *et al*. [49], with bovine serum albumin (BSA) as the standard.

#### TGF-β and TNF-α ELISA

Rat's anti-human TGF-β (catalog number, SMB100B) or TNF-α (catalog number, RTA00) monoclonal capture antibody and biotinylated detecting antibody pairs were obtained from R&D Systems (USA). ELISA was performed based on a horseradish peroxidase method described previously [50,51]. Optical density of each sample after color development was determined with a microplate reader at 450 nm.

#### Determination of 5-lipoxygenase activity in lung tissue

Lung tissues were homogenized with lysis buffer to give 50% lung lysates. Extensive dialysis (1 week) was performed on sample lysates with Tris buffer pH7.4 as dialysis buffer. Lipoxygenase activity was measured with an enzymatic colorimetric method described by Gaffney [52] using a diagnostic lipoxygenase inhibitor screening assay kit (Cayman Chemical Company, USA).

#### Hydroxyproline assay

Hydroxyproline is a modified amino acid abundant in collagen. The hydroxyproline content of the lungs was determined as a quantitative measure of collagen deposition. One half milliliter (0.5 ml) of lung homogenate was digested in 1 ml of 6N HCl for 12 hours at 110°C. Aliquots (5 μl) were then assayed with chloramine-T solution followed by development with the Erlich's reagent at 65°C for 15 minutes as previously described [53]. Absorbance was measured at 550 nm; the amount of hydroxyproline was determined against a standard curve generated with known concentrations of hydroxyproline standard.

#### Antioxidant activities and lipid peroxidation content

Lipid peroxide concentrations were determined by measuring the Malondialdahyde (MDA) end product content in plasma according to the method of Yoshioka *et al*. [54]. Reduced glutathione (GSH) estimated in the whole blood as yellow color which developed when 5, 5 dithiol-bis (2-nitrobenzoic acid) was added to sulfhydryl compounds according to the method described by Beutler *et al*. [55]. A Glutathione peroxidase (GSH-Px) activity level was assayed as described by Pigeolot *et al*. [56]. Superoxide dismutase (SOD) activity levels in the whole blood were estimated by detecting superoxide anions using nitroblue tetrazoluim formazan color development according to Minami and Yoshikawa [57]. Catalase (CAT) activity was determined according to Sinha [58].

#### Histological examination

The lungs were removed, inflated at 25 cm pressure with PBS then fixed, embedded in paraffin, sectioned and stained with hematoxylin and eosin [59]. Slides sections were examined under a light microscope.

#### Statistical analysis

SPSS (version 15) was used in data analysis. Data were analyzed with one-way analysis of variance (ANOVA) followed by a *post hoc *test (LSD alpha) for multiple comparisons. The data were expressed as mean ± standard deviation (SD). *P *values < 0.05 were considered statistically significant.

## Results and Discussion

### Effect of BA treatment on the BALF measurements in rats with lung fibrosis

Analysis of BALF in BL group without BA treatment revealed a severe inflammatory response characterized by infiltration of total cell count (neutrophils, eosinophils and other cell types) along with increases of LDH activities, protein content and lung collagen accumulation. These events are indicative of airway and/or alveolar cell damage (Table 1, 2). With the exception of the influx of eosinophils and other types of cells, BA extract fraction had promising effects on these inflammatory parameters. The anti-fibrotic action of BA may attenuate the events following the initial cell damage and the acute inflammatory phase of lung injury [60]. The present study also observed that the inflammatory responses following BL administration were attenuated by BA administration. An increased eosinophil number in BALF was reported following intratracheal BL administration, suggesting an immunological component of BL-induced pulmonary toxicity as stated by Ammon [61].

**Table 1:** BALF measurements following intratracheal in rat after different treatment conditions

Groups	LDH(U/l)	Protein (μg/ml)
Control	20.3 ± 1.04	81.4 ± 18.38
b	0.005	0.05
c	0.01	0.0001
BA	22.5 ± 3.64	99.6 ± 11.95
a	0.01	NS
b	0.0001	NS
c	0.01	0.0001
IR	28.3 ± 4.50	163.3 ± 32.22
a	NS	0.005
c	NS	0.05
BA+IR	38.1 ± 7.62	109.2 ± 15.59
a	0.05	NS
b	NS	NS
c	NS	0.0005
BL	137.2 ± 68.94	424.5 ± 32.91
a	NS	0.0001
b	NS	0.005
BA+BL	41.2 ± 5.37	150.3 ± 20.61
a	0.025	0.05
b	NS	NS
c	NS	0.005
BL+IR	155.5 ± 67.90	411.2 ± 27.37
a	NS	0.0001
b	NS	0.005
c	NS	NS
BA+BL+IR	43.7 ± 7.79	201 ± 22.17
a	0.05	0.01
b	NS	NS
c	NS	0.005

Each value represents mean ± SD.

a: Significant difference from Control

b: Significant difference from Irradiation group (IR)

c: Significant difference from BL group (BL)

NS: no significance

**Table 2:** BALF measurements following intratracheal in rat after different treatment conditions

Groups		Differential cell count (log_10_)			
	Total cell count (log_10_)	Macrophages	Neutrophils	Eosinophils	Macrophage viability (%)
Control	7.92 ± 0.55	7.56 ± 0.017	6.56 ± 0.83	6.06 ± 0.71	76.5 ± 1.21
b	NS	NS	0.05	NS	0.01
c	0.001	0.005	0.025	0.05	0.01
BA	7.74 ± 0.35	7.65 ± 0.052	5.90 ± 0.69	5.76 ± 0.16	77.0 ± 0.35
a	NS	NS	NS	NS	NS
b	0.0001	0.05	0.01	0.01	0.005
c	0.0001	0.01	0.01	0.01	0.01
IR	8.54 ± 0.104	7.46 ± 0.139	8.34 ± 0.21	6.46 ± 0.09	87.5 ± 2.25
a	NS	NS	NS	NS	NS
c	0.0001	0.005	0.05	0.025	0.025
BA+IR	8.65 ± 0.398	7.52 ± 0.069	8.51 ± 0.42	6.57 ± 0.35	81.4 ± 2.77
a	NS	NS	NS	NS	0.005
b	NS	NS	NS	NS	NS
c	0.0001	0.005	NS	0.05	0.025
BL	19.72 ± 0.346	9.93 ± 0.502	9.58 ± 0.42	8.18 ± 0.55	112.4 ± 8.31
a	0.0001	0.005	0.025	0.05	NS
b	0.0001	0.0001	0.05	0.025	0.025
BA+BL	8.6 ± 0.520	7.72 ± 0.191	9.95 ± 0.21	8.65 ± 0.40	79.7 ± 5.54
a	NS	NS	0.01	0.025	0.01
b	NS	NS	0.005	0.005	NS
c	0.0001	0.01	NS	NS	0.025
BL+IR	9.67 ± 0.346	7.50 ± 0.104	9.50 ± 0.47	7.57 ± 0.38	89.7 ± 4.16
a	0.05	NS	0.025	NS	NS
b	NS	NS	0.05	0.025	NS
c	0.0001	0.005	NS	NS	0.05
BA+BL+IR	8.95 ± 0.156	7.82 ± 0.156	8.75 ± 0.40	7.65 ± 0.55	83.5 ± 7.97
a	NS	NS	0.05	NS	0.025
b	NS	0.0001	NS	0.05	NS
c	0.0001	0.01	NS	NS	0.05

Each value represents mean ± SD.

a: Significant difference from Control

b: Significant difference from Irradiation group (IR)

c: Significant difference from BL group (BL)

NS: no significance

### Effect of BA administration on the levels of TGF-β in rats with lung fibrosis

**Figure 1:**
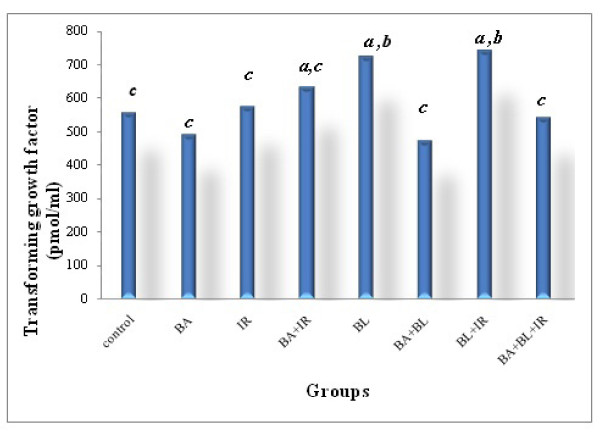
**Transforming growth factor concentration in lung tissue under different treatments conditions**. Each value represents the mean ± SD of 3 replicates. a: Significant difference from Control P = 0.05, 0.005 and 0.05 respectively. b: Significant difference from Irradiation group (IR) P = 0.025 and 0.025 respectively. c: Significant difference from BL group (BL) P = 0.005, 0.005, 0.025, 0.025, 0.01 and 0.0001 respectively.

Figure 1 shows that after intratracheal administration of BL the expression of TGF-ß1 was detected in high concentration and that BA blocked early fibrosis-related gene expression. BA administration dramatically decreased the amount of TGF-ß1-induced fibrosis observed on day 28. These findings are consistent with some previous studies [62-64] in which TGF-ß1 is also strongly associated with later stages of chronic and progressive fibrotic diseases, such as IPF [62]; TGF-ß1 auto-induction plays a part in this ongoing process [63]; and TGF-ß1 is increased in the development and the progression of radiation-induced fibrosis [64].

### Effect of BA administration on the levels of TNF-α in rat with lung fibrosis

**Figure 2:**
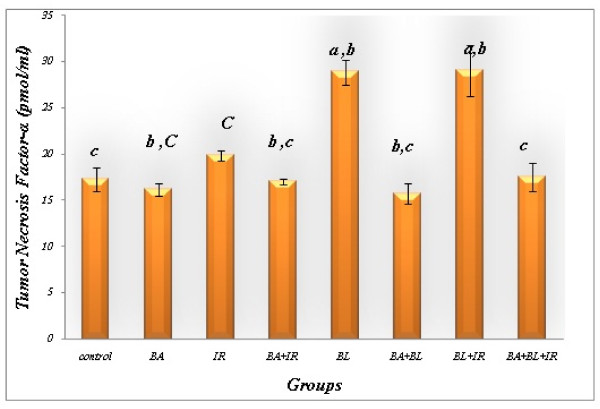
**Tumor necrosis factor-α concentration in lung of animals under different treatment conditions**. Each value represents the mean ± SD of 3 replicates. a: Significant difference from Control P = 0.005 and 0.01 respectively. b: Significant difference from Irradiation group (IR) P = 0.01, 0.005, 0.005, 0.025 and 0.025. c: Significant difference from BL group (BL) P = 0.005, 0.0005, 0.005, 0.0005, 0.005 and 0.005 respectively.

**Figure 3:**
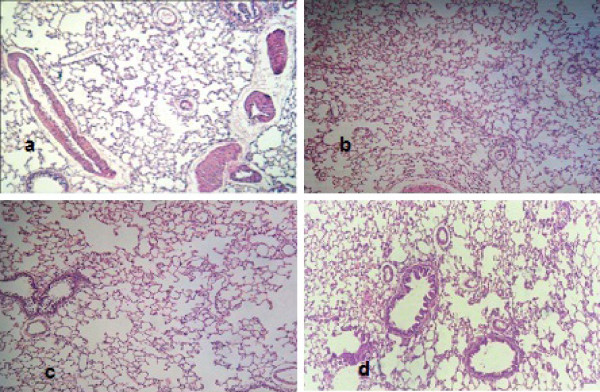
**Histopathological examination for lung in different groups (H&E X200)**. 5a control group. 5b BA group. 5c IR group. 5d BA + IR group.

**Figure 4:**
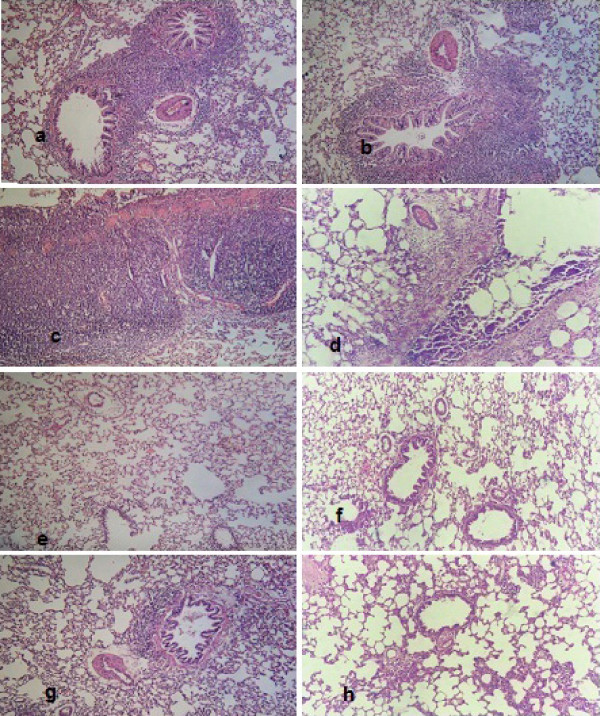
**Histopathological examination for lung in different groups (H&E X200)**. 6a & 6b BL group. 6c & 6d BL + IR group. 6e & 6f BA+BL group. 6g & 6h BA+ BL+ IR group.

Chronic overexpression of TNF-α enhanced the fibrogenic effects of BL (Figure 2). The chronic overexpression of TNF-α may be one of the factors of pulmonary fibrosis *via *modifying the immunologic reaction, increasing prostaglandin E_2 _(PGE_2_) production [39], activating matrix metalloproteinases (MMPs) or down-regulating TNF-α receptors [16]. This study strongly suggests that TNF-α can stimulate the development of pulmonary fibrosis. Down regulation of the TNF receptor 1 (TNFRI) reduced apoptosis induced by TNF-α, which may account for the accumulation of inflammatory cells (Figures 3 and 4) but may also decrease apoptosis of epithelial cells which are necessary for alveolar repair [65]. TNF-α also induces the production of PGE_2 _[66] which inhibits fibroblast proliferation [67].

### Effect of BA treatment on the histopathological findings

In the present study, the histopathological examination showed no chronic inflammation in the rat lungs. In addition, the presence of air space enlargement was observed resulting from loss or rupture of alveolar septa. However, by staining with hematoxylin and eosin stain, we found patches of fibrosis, which was in agreement with previous investigations [68,69]; the increase in hydroxyproline per lung agreed with another previous study [69]. Lung of normal rats treated with BA and or radiation exposure did not display significant differences as compared with normal animal lungs (Figures 3a, b, c and 3d). Twenty-eight days after BL instillation, rat lungs developed patchy areas of inflammation and fibrosis throughout the lung parenchyma (Figures 4a and 4b). The severity and extent of the inflammatory response to BL showed that the inflammatory process in rat lungs were characterized by an accumulation of neutrophils and lymphocytes (Figure 4a) and a predominance of lymphocytes and macrophages with few polymorphonuclear cells, as well as severe changes. Histological examination showed increased wall thickness of the pulmonary arteries (Figure 4b). Rat lungs exposed to both BL and irradiation (BL+IR) showed diffuse, heavy infiltration of inflammatory cells and cystic changes (Figures 4c and 4d). Lung of a BA+BL treated group rats received repeated high dose of BA seven days before intratracheal instillation of BL to the end of the experimental phase of the study showed thin interalveolar septa, a lack of inflamed cells and normal-appearing bronchioles and alveolar ducts (Figures 4e and 4f). Histopathological examination in the group of rats receiving BA+BL+IR showed fewer fibrotic lesions and local infiltrations of inflammatory cells (Figures 4g and 4h).

### Effect of BA administration on the activity levels of 5-LOX in lung of rats bearing fibrosis

**Figure 5:**
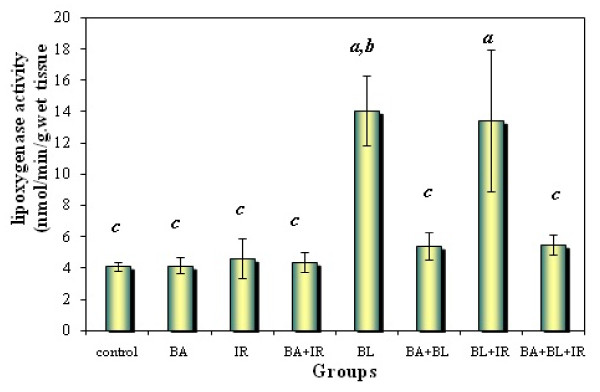
**5-Lipoxygenase activity levels in lung tissue of animals under different treatments conditions**. Each value represents the mean ± SD of 3 replicates. a: Significant difference from Control P = 0.0005 and 0.05 respectively. b: Significant difference from Irradiation group (IR) P = 0.0005. c: Significant difference from BL group (BL) P = 0.005, 0.005, 0.005, 0.005, 0.005 and 0.005 respectively.

Animals subjected to BL had higher pulmonary 5-LOX (Figure 5). Figures 1, 2 and 5 demonstrate that BA, administered orally, inhibited the TGF-ß1 and TNF-α as well as the activity level of 5-LOX enzymes. 5-LOX was suggested to be a target of BA [70,21]; and leukotrienes (LT) are potent mediators of inflammation; thus, suppression of LT formation by BA may be the underlying mechanism of the anti-inflammatory actions of BA.

### Effect of BA administration on the antioxidant status in rats with lung fibrosis

BA have been successfully used to prevent injury responses in activated macrophages [71] and are effective agents in preventing oxidant-induced injury responses [72]. Activated leukocytes can release reactive oxygen and nitrogen species (superoxide, hydrogen peroxide, hydroxyl radical, hypochlorous acid, nitric oxide and peroxynitrite) and proteases that sustain the injury/repair processes thought to contribute to the fibrotic processes [73]. BA scavenged a broad spectrum of reactive oxygen and nitrogen species (Tables 3, 4 and 5). This scenario suggests an approach to breaking the feedback loop by using catalytic antioxidants BA.

**Table 3:** Reduced glutathione concentration and glutathione perosidase activity in RBCs compacted cells and lung tissue homogenate in rat after different treatment conditions

Parameters Groups	GSH		GSH-Px	
	Blood (mg/dl packed RBCs)	Lung (μg/g tissue)	Blood (μg oxidized GSH/ min/ml packed RBCs)	Lung (μg oxidized GSH/ min/g tissue)
Control	188.00 ± 8.35	206.1 ± 4.20	272.52 ± 12.41	465.6 ± 35.83
b	NS	NS	NS	NS
c	P = 0.0001	P = 0.0001	P = 0.0001	P = 0.001
BA	238.91 ± 23.48	199.3 ± 4.93	281.78 ± 19.89	441.8 ± 52.42
a	P = 0.0001	NS	NS	NS
b	P = 0.0001	NS	NS	NS
c	P = 0.0001	P = 0.0001	P = 0.0001	P = 0.004
IR	175.03 ± 45.50	202.4 ± 4.48	293.93 ± 15.70	416.8 ± 19.32
a	NS	NS	NS	NS
c	P = 0.0001	P = 0.0001	P = 0.0001	P = 0.029
BA +IR	177.31 ± 27.77	209.5 ± 8.23	286.04 ± 20.82	458.1 ± 27.81
a	NS	NS	NS	NS
b	NS	NS	NS	NS
c	P = 0.0001	P = 0.0001	P = 0.0001	P = 0.001
BL	117.07 ± 10.01	169.9 ± 1.71	175.89 ± 26.47	353.9 ± 40.76
a	P = 0.0001	P = 0.0001	P = 0.0001	P = 0.001
b	P = 0.0001	P = 0.0001	P = 0.0001	P = 0.029
BA + BL	187.17 ± 13.54	191.9 ± 1.29	195.26 ± 18.53	391.9 ± 21.37
a	NS	P = 0.001	P = 0.0001	P = 0.012
b	NS	P = 0.012	P = 0.0001	NS
c	P = 0.0001	P = 0.0001	NS	NS
BL + IR	187.17 ± 13.54	201. 4 ± 4.07	195.26 ± 18.53	437.5 ± 21.18
a	NS	NS	P = 0.0001	NS
b	NS	NS	P = 0.0001	NS
c	P = 0.0001	P = 0.0001	NS	P = 0.006
BA + BL + IR	161.77 ± 15.77	191.0 ± 3.70	280.07 ± 19.41	411.3 ± 21.01
a	NS	P = 0.001	NS	NS
b	NS	P = 0.007	NS	NS
c	P = 0.002	P = 0.0001	P = 0.0001	P = 0.043

Each value represents mean ± SD (*n *= 10).

n = Number of tests

a: Significant difference from Control

b: Significant difference from Irradiation group (IR)

c: Significant difference from BL group (BL)

NS: no significance

**Table 4:** Superoxide dismutase and catalase activity levels in RBCs compacted cells and lung tissue homogenate in rat after different treatment conditions

Parameters Groups	SOD		Cat	
	Blood (g/ml packed RBCs)	Lung (μg/g wet tissue)	Blood (μmol/ml packed RBCs	Lung (μmol/g wet tissue)
Control	5.065 ± 0.51	7.366 ± 0.60	398.425 ± 40.57	165.183 ± 18.10
b	NS	NS	NS	NS
c	P = 0.0001	P = 0.01	NS	p = 0.009
BA	5.356 ± 0.47	7.307 ± 1.06	389.510 ± 36.72	162.477 ± 11.25
a	NS	NS	NS	NS
b	NS	NS	NS	NS
c	P = 0.0001	P = 0.014	NS	P = 0.018
IR	4.872 ± 0.53	7.233 ± 0.43	377.941 ± 51.08	166.333 ± 13.41
a	NS	NS	NS	NS
c	P = 0.0001	P = 0.019	NS	P = 0.007
BA +IR	4.875 ± 0.50	6.850 ± 0.84	404.467 ± 12.32	154.373 ± 10.08
a	NS	NS	NS	NS
b	NS	NS	NS	NS
c	P = 0.0001	NS	NS	NS
BL	3.064 ± 0.28	5.973 ± 0.28	382.335 ± 56.22	141.187 ± 1.31
a	P = 0.0001	P = 0.01	NS	p = 0.009
b	P = 0.0001	P = 0.019	NS	P = 0.007
BA + BL	4.805 ± 0.56	7.097 ± 0.29	434.126 ± 45.83	171.960 ± 4.12
a	NS	NS	NS	NS
b	NS	NS	p = 0.026	NS
c	P = 0.0001	P = 0.033	p = 0.039	P = 0.002
BL + IR	4.805 ± 0.56	6.970 ± 0.21	434.126 ± 45.83	157.110 ± 4.84
a	NS	NS	NS	NS
b	NS	NS	p = 0.026	NS
c	P = 0.0001	NS	p = 0.039	NS
BA + BL + IR	4.342 ± 0.77	6.757 ± 0.47	448.533 ± 32.19	181.187 ± 2.17
a	P = 0.025	NS	p = 0.046	NS
b	NS	NS	p = 0.006	NS
c	P = 0.0001	NS	p = 0.009	P = 0.0001

Each value represents mean ± SD (*n *= 10).

n = Number of tests

a: Significant difference from Control

b: Significant difference from Irradiation group (IR)

c: Significant difference from Bleomycin group (BL)

NS: no significance

**Table 5:** Lipid peroxide concentration in plasma and lung tissue homogenate of rat under different treatment conditions

	LP	
Groups	Blood (μg/ml plasma)	Lung (μg/g tissue)
Control	106.06 ± 17.10	242.20 ± 18.20
b	NS	NS
c	P = 0.0001	P = 0.0001
BA	103.93 ± 15.73	245.75 ± 10.63
a	NS	NS
b	NS	NS
c	P = 0.0001	P = 0.001
IR	105.55 ± 14.39	241.31 ± 16.51
a	NS	NS
c	P = 0.0001	P = 0.0001
BA +IR	101.04 ± 9.68	247.08 ± 10.01
a	NS	NS
b	NS	NS
c	P = 0.0001	P = 0.001
BL	143.66 ± 11.41	287.75 ± 11.28
a	P = 0.0001	P = 0.0001
b	P = 0.0001	P = 0.0001
BA + BL	111.71 ± 10.99	267.97 ± 9.82
a	NS	P = 0.019
b	NS	P = 0.016
c	P = 0.0001	NS
BL + IR	99.31 ± 6.78	263.75 ± 6.99
a	NS	P = 0.044
b	NS	P = 0.037
c	P = 0.0001	P = 0.027
BA + BL + IR	105.49 ± 9.22	263.31 ± 8.74
a	NS	P = 0.048
b	NS	P = 0.040
c	P = 0.0001	P = 0.025

Each value represents mean ± SD (*n *= 10).

n = Number of tests

a: Significant difference from Control

b: Significant difference from Irradiation group (IR)

c: Significant difference from BL group (BL)

NS: no significance

### Effect of radiation exposure on the BALF measurements and TGF-β1 and TNF-α in rats with lung fibrosis

Sublethal and lethal lung irradiation can trigger a number of genetic and molecular events that can have both short-term effects and longer-term effects [74]. Some more immediate effects include recruitment of inflammatory cells to lung, up-regulation of adhesion molecules, induction of oxidative injury and generation of a number of pro-inflammatory cytokines and chemokines, notably TNF-α [75-79]. The present study demonstrates the effect of radiation exposure on the lung syndrome of fibrosis formation (Table 1, 2). Surprisingly, lung irradiation had no significant changes in LDH activity level, total protein, neutrophils and eosinophils while macrophages viability percentage recorded *P < 0.05 *on levels of inflammatory cells or acute inflammatory mediators measured in BALF of BL exposed animals. Radiation pneumonitis and subsequent lung fibrosis in rats were not detectable with the radiation dose given in this study (0.5 Gy/week for two week). Nonetheless, the capacity of the lung to mount both acute inflammatory and subsequent fibrotic responses was diminished. Our results (Table 1, 2) suggest that inflammatory cells, a significant source of the measured inflammatory mediators, had appropriate basal function at the early time points in this study. However, the response to BL administration was significantly altered in rats. The mechanism for these effects is not clear.

### Effect of radiation exposure on the antioxidant status in rats with lung fibrosis

Oxidative stress and inflammation are related. In the initial inflammation, immune cells migrate to inflammatory sites and release various proinflammatory cytokines that function in a coordinative manner to commence an inflammatory cascade and resolve this acute inflammation [80]. The aberrations in the apoptosis and phagocytosis of *in situ *inflammatory cells may lead to an unresolved chronic inflammation [81]. In a setting of chronic inflammation, the persistent tissue damage and cell proliferation are associated with excessive of reactive oxygen and nitrogen species [82].

In the present study, fibrosis in rat lungs induced significant deleterious changes in antioxidant status with or without whole body irradiation. The results recorded a significant increase in lipid peroxidation level, a marked depletion in GSH content and decline in SOD and GSH-Px activity in both the blood and lung tissues of the animals treated with BA.

### Possible role of BA treatment for augmentation and regulation of antioxidant status

5-LOX must co-localize with 5-LOX activating protein and cytosolic phospholipase A2 then redistribute to the nuclear membrane to perform its action [83]. It plays a central role in cellular leukotriene synthesis. 5LO converts arachidonic acid, released from the membranes by phospholipase A2, into 5(S)-hydroperoxy-6, 8, 11, 14-eicosatetraenoic acid (5-HPETE), and subsequently into the epoxide intermediate leukotriene A4 (LTA4). Hydrolysis of LTA4 by LTA4 hydrolase leads to the formation of the potent neutrophil chemoattractant LTB4, whereas conjugation of LTA4 with glutathione through the action of LTC4 synthase yields LTC4, which then is sequentially degraded into LTD4 and LTE4. The cysteinyl- leukotrienes, which constitute slow-reacting substance of anaphylaxis, are known to contract airway smooth muscle, increase vascular permeability, and promote mucus secretion. Werz [84] suggested that 5-LOX activity was regulated by both cellular redox tone and enzyme phosphorylation which could affect its redistribution. Thus, 5-LOX inhibitors such as BA may act on either redox mechanism or enzyme distribution.

**Figure 6:**
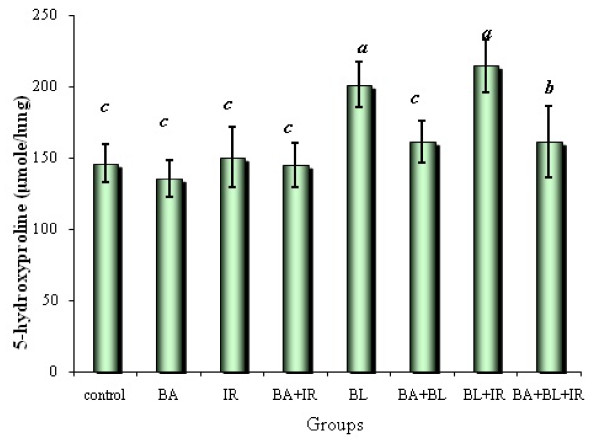
**Hydroxyproline concentration in lung of rats under different treatments conditions**. Each value represents the mean ± SD of 3 replicates. a: Significant difference from Control P = 0.025 and 0.005 respectively. b: Significant difference from Irradiation group (IR) P = 0.025. c: Significant difference from BL group (BL) P = 0.025, 0.005, 0.05, 0.025 and 0.05 respectively.

A balance between oxidants and antioxidants is a prerequisite for normal lung homeostasis. Induction of these antioxidant enzymes and related proteins after pulmonary insults may protect the lung and promote normal repair. These enzymes include SOD, catalase and glutamate content, the rate-limiting enzyme in glutathione synthesis [85]. The primary antioxidant enzymes in the extracellular matrix and alveolar lining fluid that may inhibit oxidative activation are extracellular glutathione peroxidase [86] and SOD [87]. Table 3, 4 shows the augmentation of the levels of glutathione level, SOD and catalase activity in the blood and lung of the fibrotic rat with BA treatment. The role of BA in amelioration of TGF-ß1 experrsion may be the route of BA to modulate the antioxidant status. TGF-ß1 has the main role in fibrosis in lung diseases. TGF-ß1 stimulates accumulation of extracellular matrix through increased transcription of collagen mRNA [88], thereby leading to the accumulation of collagen content represented by the increase of the levels of 5-hydroxyproline concentration as a quantitative measure of collagen deposition (Figure 6). Thus, consistently elevated levels of TGF-ß1 in the lung may serve as a stimulus for myofibroblast activation and production of extracellular matrix.

There are several potential interactions between TGF-ß1 and oxidants/antioxidants in the lung. For example, TGF-ß1 differentiated myofibroblasts can themselves serve as a source of oxidant production [89]. *In vitro *studies have shown that ROS increases the release of TGF-ß1 from pulmonary epithelial cells [90]. TGF-ß1 down regulates the mRNA synthesis of glutamate cysteine ligase, the rate-limiting enzyme in the production of the antioxidant molecule glutathione [91]. Glutathione synthesis is decreased in TGF-ß over expressing mice [92]. Regulation of TGF-ß1 is responsible for the amelioration of the antioxidant status.

During the inflammatory processes which play a critical role in lung fibrosis, 5-LOX, an enzyme involved in the oxygenation of the arachidonic acid, is upregulated in lung fibrosis pathologies [93]. In the present study, the administration of BA which is a 5-LOX inhibitor reversed the fibrosis-related increase in oxidative damage (Tables 3, 4 and 5). This confirms the suggested role of 5-LOX in lung fibrosis.

## Conclusion

BA attenuate the BL-induced injury response in rats, such as collagen accumulation, airway dysfunction and injury. This study suggests that the blocking of 5-LOX may prevent the progression of fibrosis.

## Abbreviations

BA: Boswellic acids; BL: Bleomycin; NS: normal saline; TGF- β1: transforming growth factor-β1; IR: exposed to γ irradiation; LDH: lactate dehydrogenase activity levels; TNF-α: tumor necrosis factor-α; 5-LOX: 5-lipoxygenase enzyme activity; GSH-Px: glutathione peroxidase; BALF: bronchoalveolar lavage fluid; GSH: reduced glutathione; IPF: Idiopathic pulmonary fibrosis; SOD: superoxide dismutase; CAT: catalase; LP: lipid peroxidation; MDA: malondialdahyde; ECM: extracellular matrix; MMPs: matrix metalloproteinases; LT: prostaglandin E_2 _(PGE_2_) and leukotrienes.

## Competing interests

The authors declare that they have no competing interests.

## Authors' contributions

EN designed the study, supervised the experiments and wrote the manuscript. SZM performed the animal experiments and prepared BA. Both authors supervised the research assistants to carry out clinical chemistry assays. Both authors read and approved the final version of the manuscript.
